# Exposure to Methylmercury at Juvenile Stage Worsens Autism-Like Symptoms in Adult BTBR T+tf/J Mice Due to Lack of Nuclear Factor Erythroid 2-Related Factor 2 Signaling Upregulation in Periphery and Brain

**DOI:** 10.3390/toxics11060546

**Published:** 2023-06-20

**Authors:** Mohammad M. Algahtani, Sheikh F. Ahmad, Layla A. Alkharashi, Naif O. Al-Harbi, Wael A. Alanazi, Abdullah S. Alhamed, Sabry M. Attia, Saleh A. Bakheet, Khalid E. Ibrahim, Ahmed Nadeem

**Affiliations:** 1 Department of Pharmacology and Toxicology, College of Pharmacy, King Saud University, Riyadh 11451, Saudi Arabia; 2 Department of Zoology, College of Science, King Saud University, Riyadh 11451, Saudi Arabia

**Keywords:** antioxidants, autism spectrum disorder, methylmercury chloride, Nrf2 signaling, oxidative stress, neutrophils

## Abstract

Autism spectrum disorder (ASD) is a multifaceted developmental condition that first appears in infancy. The condition is characterized by recurrent patterns in behavior and impairments in social and vocalization abilities. Methylmercury is a toxic environmental pollutant, and its derivatives are the major source of organic mercury to human beings. Inorganic mercury, which is released from a variety of pollutants into oceans, rivers, and streams, is transformed into methylmercury by bacteria and plankton in the water, which later builds up in fish and shellfish, and then enters humans through the consumption of fish and shellfish and increases the risk of developing ASD by disturbing the oxidant–antioxidant balance. However, there has been no prior research to determine the effect of juvenile exposure of methylmercury chloride on adult BTBR mice. Therefore, the current study evaluated the effect of methylmercury chloride administered during the juvenile stage on autism-like behavior (three-chambered sociability, marble burying, self-grooming tests) and oxidant–antioxidant balance (specifically Nrf2, HO-1, SOD-1, NF-kB, iNOS, MPO, and 3-nitrotyrosine) in the peripheral neutrophils and cortex of adult BTBR and C57BL/6 (B6) mice. Our results show that exposure to methylmercury chloride at a juvenile stage results in autism-like symptoms in adult BTBR mice which are related to a lack of upregulation of the Nrf2 signaling pathway as demonstrated by no significant changes in the expression of Nrf2, HO-1, and SOD-1 in the periphery and cortex. On the other hand, methylmercury chloride administration at a juvenile stage increased oxidative inflammation as depicted by a significant increase in the levels of NF-kB, iNOS, MPO, and 3-nitrotyrosine in the periphery and cortex of adult BTBR mice. This study suggests that juvenile exposure to methylmercury chloride contributes to the worsening of autism-like behavior in adult BTBR mice through the disruption of the oxidant–antioxidant balance in the peripheral compartment and CNS. Strategies that elevate Nrf2 signaling may be useful to counteract toxicant-mediated worsening of ASD and may improve quality of life.

## 1. Introduction

Autism spectrum disorder (ASD) is a neurodevelopmental disorder which is associated with immune dysfunction. The common repercussions of the disease include difficulty in social communication, lack of interest, and deviations in normal behavior which makes this quite challenging in terms of the burden on society and country [1]. Since 1996, prevalence studies have been conducted in more than 15 countries to determine the increasing number of patients with autism spectrum disorder. The results showed that there were 4.1 subjects with ASD for every 10,000 people in the UK in 1966 whereas it increased to 13 subjects with ASD for every 10,000 people in 2014, and this figure is expected to rise substantially in the future [2,3]. Compared to females, ASD affects males more with a ratio of 1:3, and this disorder is quite prevalent in developed as well as low- and middle-income nations [4]. Numerous studies reported that the United States, Sweden, and Denmark are the leading countries with the highest prevalence of autism and the highest incidence of ASD [5,6]. The continuous increase in patients with autism will automatically increase the global autism mortality index, which will have an adverse economic and health burden on the USA (USD 268 billion in 2015), and this figure is expected to rise by USD 460 billion by 2050 [7]. The global increase in the autism index suggests the need to seek therapeutic treatment for the management of the disease. Although many investigations have been conducted worldwide to find out the cellular and molecular mechanisms of the disease, the exact mechanism leading to the development/progression of ASD remains elusive.

Mercury is considered one of the major environmental pollutants whose exposure is found to be toxic in humans [8,9]. Mercury is present in three chemical forms i.e., HgO, organic, and inorganic mercury. Methylmercury, an organic mercury compound, causes deleterious effects in humans and animals, including neurological behavioral disorders [9,10]. Previous studies have shown that the effects of exposure to mercury compounds during prenatal/postnatal/adulthood stage cause the development of autism-like symptoms in mice through various mechanisms that include the expression of pro-inflammatory cytokines and altered transcription factor signaling in CNS [11,12,13]. However, the effect of juvenile exposure of methylmercury chloride on autism-like behavior in adult BTBR and B6 mice remains to be explored, especially with respect to oxidant–antioxidant balance.

Reactive oxygen species (ROS), a major cause of cell damage, are known to result from metal oxidation as well as other sources. The presence of high levels of unsaturated lipids and the rapid rate of oxidative metabolism increases the risk of oxidative damage in the brain [14]. The mechanism of action of mercury and its analogs in promoting oxidative stress in the brain and other tissue has been extensively studied over the past years. For instance, a study showed that the administration of mercury chloride at a dose of 0.375 mg for 45 consecutive days promoted the development of neurodegenerative disorders by attenuating the level of antioxidant enzymes and increasing lipid peroxidation in the motor cortex of adult rats [15]. Another study showed that exposure to mercury vapor (1 mg) in adult female Sprague-Dawley rat for 11 consecutive days (2 h each day) aided in the elevation of oxidative stress in the cortex region of the brain followed by the deposition of mercury in the brain and kidney tissues [16]. In another study, the depletion of antioxidants led to an increase in oxidative enzymes such as NADPH oxidase/iNOS and lipid peroxidation in periphery/CNS which was linked to an increase in autism-like symptoms in BTBR mice [17]. However, the role of juvenile exposure of methylmercury chloride on Nrf2 signaling and oxidative stress in the peripheral neutrophils and cortex in adult BTBR and B6 mice needs to be elucidated.

Master redox transcription factor known as nuclear factor erythroid-2 related factor (Nrf2) plays an important function against oxidative stress by regulating the cellular defense mechanisms. Dysregulation in Nrf2 signaling is mainly oxidative stress-associated immune and neurological disorders including ASD [17,18]. For instance, several studies have shown that ASD subjects and animals with autism-like behavior display a decrease in Nrf2 expression [19,20,21]. In this study, we intended to examine the effect of methylmercury chloride on Nrf2-mediated signaling in the cortex of BTBR and B6 mice. To our knowledge, this is the first study to look at how exposure to methylmercury chloride at a juvenile stage affects the autism-like behavior and oxidant–antioxidant balance in the periphery and brain of adult BTBR and B6 mice. Our findings highlight that the exposure to methylmercury chloride during the juvenile stage aggravated autism-like behavior in adult BTBR mice probably through a lack of Nrf2-mediated antioxidant protection in the periphery and CNS.

## 2. Materials and Methods

### 2.1. Animal Model

The male C57BL/6 (B6) and BTBR T+ Itpr3tf/J (BTBR) mice were obtained from Jackson Laboratory in Bar Harbor, ME, USA. The mice were bred in the department’s animal care facility and were kept in specific pathogen-free and proper hygienic conditions and a controlled environment of temperature/humidity, and a 12 h light–dark cycle. Mice had unrestricted access to food and water. The Animal Care and Research Committee of the College of Pharmacy, King Saud University, approved all animal experiments. BTBR mice are well known for showing autism-like behavior spontaneously which has been studied in detail by multiple investigators in the past. BTBR mice show socialization defects, and stereotypical repetitive behavioral patterns similar to human ASD subjects which can be studied through a battery of behavioral tests as described below [17,19,21].

### 2.2. Drug Administration

**Figure 1:**
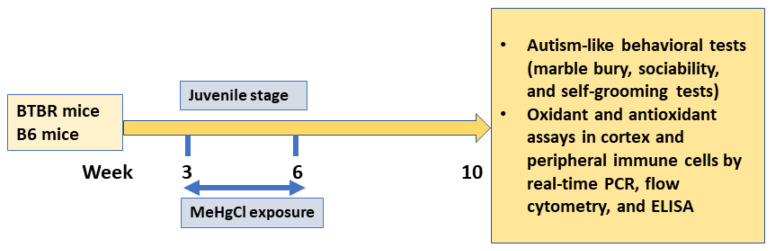
Experimental design.

To examine the impact of the environmental contaminant methylmercury chloride (MeHgCl) on autism-like symptoms in BTBR mice, they were administered with MeHgCl at a dose of 0.3 ppm in their drinking water for a duration of three weeks, starting at the age of 3 weeks [22]. At the age of 6 weeks, the mice were switched to normal tap water for a period of 4 weeks followed by behavioral tests in week 10. The mice were allocated randomly to four groups: (1) B6 mice treated with a vehicle (control group), (2) B6 mice treated with MeHgCl, (3) BTBR mice treated with a vehicle, and (4) BTBR mice treated with MeHgCl. The vehicle in this study is normal drinking water which was provided to either B6 mice (group 1) or BTBR mice (group 3) throughout the study. Behavioral assessments, including the marble-burying test, self-grooming test, and three-chambered social interaction test, were conducted in week 10 (Figure 1).

### 2.3. Marble Burying Test

The marble-burying behavior test, as described earlier, was used to assess autism-like behavior [17,23]. Mice were placed on top of unperfumed bedding containing 20 marbles arranged in a 4 *×* 5 grid. After 30 min of free exploration, an independent researcher, unaware of the experimental conditions, counted the number of marbles buried by each mouse. If greater than 50% area of the marble was covered by the bedding, it was considered buried.

### 2.4. Self-Grooming Test

The spontaneous self-grooming test, described in previous studies, was conducted to measure autism-like behavior [17,23,24]. Mice were placed in an empty cage (dimension of 27 × 17 × 13 cm) for a 10 min habituation period, followed by a 10 min observation period. An independent observer, blinded to the experimental conditions, recorded the total time spent by each mouse engaged in self-grooming behavior which included head washing, body/tail/genital grooming, and paw and leg licking.

### 2.5. Three-Chambered Sociability Test

The three-chambered sociability test, as described in earlier studies, was used to assess social behavior [17,23]. Mice were habituated to a three-chambered rectangular acrylic box (22 cm × 60 cm × 22 cm) and allowed to explore for 10 min. After acclimatization to the test surroundings, the test mouse was placed again in the acrylic test box for exploration of all three chambers for a period of 10 min. Social interaction time was defined as the time spent by the test mouse in interactions/the vicinity with a novel mouse and it was observed and recorded by two independent scientists blinded to the treatment conditions.

Following the completion of the behavioral tests, the mice were euthanized for further biochemical and molecular analyses. Tissue samples (spleen/brain) were collected for subsequent molecular/biochemical analyses.

### 2.6. Flow Cytometry

A single-cell suspension was created from splenic tissue using established protocols outlined in previous studies for flow cytometry analysis [17,25]. To label the leukocytes in the splenic cell suspension, cell surface monoclonal antibodies against GR-1 were used, which were conjugated to FITC, APC, or APC-Cy7 [Biolegend (San Diego, CA, USA) and Santa Cruz Biotech (Dallas, TX, USA)]. Subsequently, permeabilization and fixation procedures were conducted to allow for intracellular staining. Leukocytes were labeled with specific monoclonal antibodies conjugated to FITC, PE, or APC against intracellular proteins including SOD1, Nitrotyrosine, Nrf2, p-NFkB, and iNOS [Biolegend (San Diego, CA, USA), Cell Signaling Tech (Danvers, MA, USA), and Santa Cruz Biotech (Dallas, TX, USA)]. Flow cytometry was then performed, acquiring 10,000 events for each sample, and the resulting data were analyzed using Cytomics FC500 software from Beckman Coulter (Brea, CA, USA), following the methodology described in previous reports [26,27].

### 2.7. Trans-Activation ELISA Assay in the Cortex for Nrf2 Binding with Its Antioxidant Response Element

To assess the Nrf2 trans-activation binding activity to the antioxidant response element (ARE) in the cortex, the Trans-AM Nrf2 kit from Active Motif (Carlsbad, CA, USA) was employed, following the methodology outlined in the previous study [25]. The assay aimed to determine the binding activity of Nrf2 to its specific ARE sequence, which is 5′-GTCACAGTACTCAGCAGAATCTG-3′. The binding activity was quantified by measuring the generation of a colored product at 450 nm, which is indicative of the specific Nrf2 activity in the nuclear extracts. The data obtained from the assay were presented as fold differences, representing the relative change in Nrf2 binding activity compared to the control.

### 2.8. Real-Time PCR

To evaluate mRNA expression in the cortex, the High-Capacity cDNA Archive Kit from Applied Biosystems (Waltham, MA, USA) was utilized for the reverse transcription of RNA into cDNA, following established protocols described in previous studies [17,27]. Gene expression analysis was performed using the ABI PRISM 7500 Sequence Detection System from Applied Biosystems. For the real-time PCR analysis of specific genes, iNOS, NFkBp65, SOD1, HO-1, Nrf2, and GAPDH primers were used (GenScript primers, Piscataway, NJ, USA). The relative gene expression levels in different cortical brain samples were determined using the comparative delta-delta Ct method, enabling the calculation of fold changes in gene expression compared to the control sample.

### 2.9. Lipid Peroxides Measurement in Brain

The assessment of lipid peroxides in cortical brain samples followed an established protocol outlined in the study [17]. The concentration of lipid peroxides was measured and presented in nmol/mg protein.

### 2.10. Myeloperoxidase (MPO) Activity Measurement in Brain

To evaluate myeloperoxidase (MPO) activity in the cortex samples as an indicator of neutrophil inflammation, a method described earlier was employed [25]. The supernatants obtained from the cortex samples were mixed with MPO substrate buffer containing O-dianisidine (0.167 mg/mL) and H_2_O_2_ (0.0005%) in 50 mM potassium phosphate buffer. Following a 20 min incubation at 25 °C, the MPO activity was determined by measuring the absorbance at 450 nm using a microplate reader.

### 2.11. Data Analysis

The data were presented as mean ± SEM, and statistical values were considered significant at *p* < 0.05. The statistical analysis was performed using GraphPad Prism 9 software (GraphPad Software, San Diego, CA, USA). A two-way ANOVA was conducted followed by Tukey’s post-hoc test for multiple comparisons to assess the differences and interactions among different groups.

## 3. Results

### 3.1. Effect of Methylmercury Chloride Administration during Juvenile Stage on Autism-like Behavior in Adult BTBR and B6 Mice

**Figure 2:**
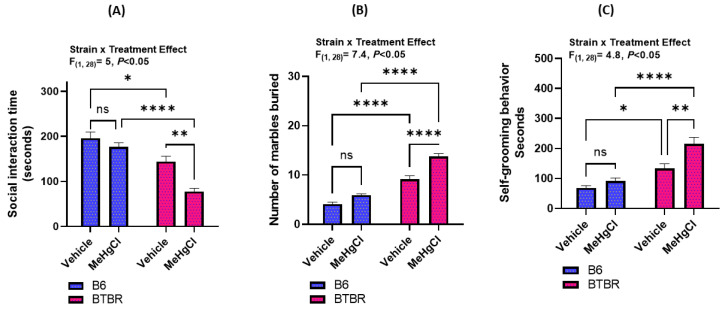
Effect of methylmercury chloride (MeHgCl) on autism-like behavioral parameters in B6 (social strain) and BTBR (asocial strain) mice. (**A**) Social interaction time, (**B**) marble burying test and (**C**) self-grooming test. Values are shown as mean ± SEM; n = 8/group. * *p* < 0.05; ** *p* < 0.01; **** *p* < 0.0001; ns = not significant.

Our study investigated the potential long-term effects of juvenile exposure to methylmercury chloride (MeHgCl) on adult mice, specifically in relation to autism-like behavioral parameters. To assess this, we examined several behavioral parameters such as the time spent near a novel mouse, the number of marbles buried, and self-grooming behavior in both B6 (a social strain) and BTBR (an asocial strain) mice. Our results show that exposure to methylmercury chloride during the juvenile period aggravated autism-like behavioral disturbances in adult BTBR mice. Briefly, the effect of methylmercury chloride on the autism-like behavioral tests in B6 and BTBR mice was investigated using a variety of experiments such as three-chambered sociability, marble bury test, and self-grooming behavior (Figure 2). Upon receiving methylmercury chloride treatment in the juvenile stage, BTBR mice in adulthood had decreased social communication skills (i.e., spent less time in the vicinity of the novel mouse) than vehicle-treated BTBR mice during the same juvenile period (Figure 2A). Furthermore, methylmercury chloride-treated BTBR mice displayed increased marble-burying behavior as compared to methylmercury chloride-treated B6 mice with a statistically significant *p*-value < 0.005 (Figure 2B). In addition, methylmercury chloride-treated BTBR mice spent more time in self-grooming behaviors as compared to methylmercury chloride-treated B6 mice, indicating the exacerbation of autism-like behavior in methylmercury chloride-treated BTBR mice (Figure 2C). However, B6 mice receiving methylmercury chloride during the juvenile period also displayed minor symptoms of autism-like behavioral disturbances than vehicle-treated B6 mice; however, these effects were not significant in social communication, marble burying, and self-grooming tests (Figure 2A–C). These findings together depict that juvenile exposure to methylmercury chloride may increase autism-like behavior in adult BTBR mice.

### 3.2. Effect of Methylmercury Chloride during Juvenile Stage on Nrf2-Mediated Signaling in Peripheral Neutrophils of Adult BTBR and B6 Mice

**Figure 3:**
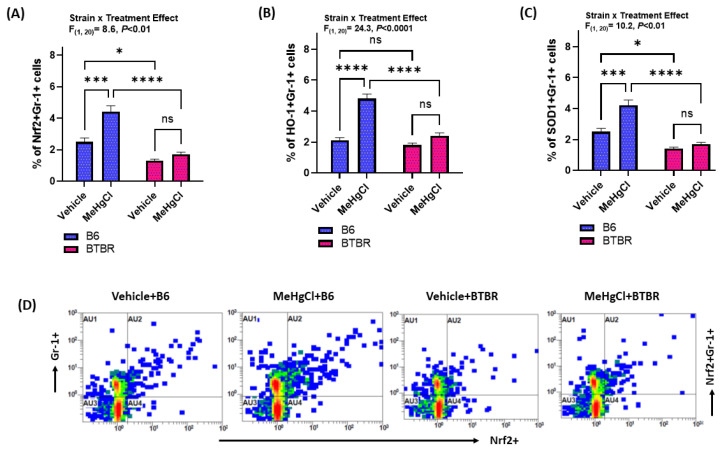
Effect of methylmercury chloride on Nrf2-mediated signaling in peripheral neutrophils of B6 and BTBR mice. (**A**) % of Nrf2 expression in Gr-1+ cells, (**B**) % of HO-1 expression in Gr-1+ cells, (**C**) % of SOD-1 expression in Gr-1+ cells, and (**D**) Representative flow plot demonstrating double immunostaining of Nrf2+Gr-1+ cells. Values are shown as mean ± SEM; n = 6/group. * *p* < 0.05; *** *p* < 0.001; **** *p* < 0.0001; ns = not significant.

After studying the effect of juvenile exposure to methylmercury chloride on autism-like symptoms, the effect on Nrf2 and antioxidant enzymes was examined in the peripheral neutrophils (Gr-1+ cells) of B6 and BTBR mice. Our results indicate that exposure to methylmercury chloride during their juvenile stage leads to a lack of Nrf2 upregulation in the neutrophils of adult BTBR mice (Figure 3A). On the contrary, methylmercury chloride-treated B6 mice had upregulated neutrophilic Nrf2 signaling in adulthood as depicted by increased Nrf2+Gr-1+ cells (Figure 3A,D). In addition, BTBR mice receiving methylmercury chloride treatment during the juvenile stage did not have elevated heme-oxygenase-1 (HO-1) expression in Gr-1+ cells, whereas B6 mice exposed to methylmercury chloride treatment had a higher expression of HO-1 in Gr-1+ cells than their respective control group (Figure 3B). Similarly, BTBR mice receiving methylmercury chloride treatment during the juvenile stage did not have elevated SOD-1 in Gr-1+ cells, whereas B6 mice exposed to methylmercury chloride treatment had a higher expression of SOD-1 in Gr-1+cells than their respective control group (Figure 3C). These findings suggested that a lack of Nrf2 upregulation in peripheral neutrophils may intensify autism-like symptoms in adult BTBR mice subjected to methylmercury chloride treatment during the juvenile period.

### 3.3. Effect of Methylmercury Chloride during Juvenile Stage on NF-kB-Mediated Signaling in Peripheral Neutrophils of Adult BTBR and B6 Mice

**Figure 4:**
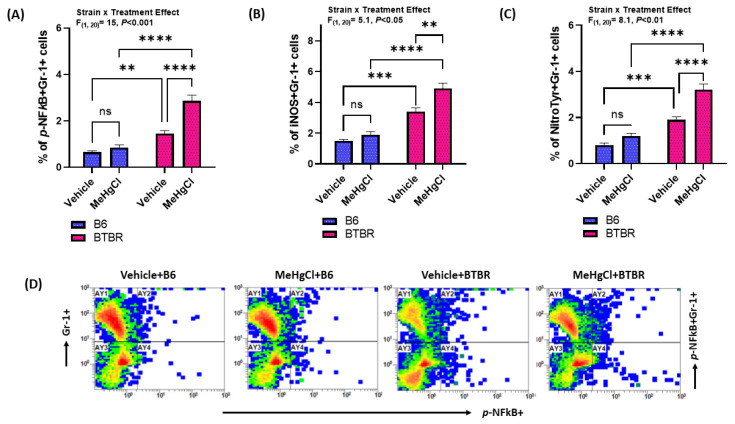
Effect of methylmercury chloride on NFkB-mediated signaling in peripheral neutrophils of B6 and BTBR mice. (**A**) % of p-NFkB expression in Gr-1+ cells, (**B**) % of iNOS expression in Gr-1+ cells, (**C**) % of Nitrotyrosine expression in Gr-1+ cells, and (**D**) Illustrative flow plot for p-NFkB+Gr-1+ double immunostaining. Values are shown as mean ± SEM; n = 6/group. ** *p* < 0.01; *** *p* < 0.01; **** *p* < 0.0001; ns = not significant.

The expression of NF-kB and oxidative stress markers (i.e., iNOS, Nitrotyrosine) was analyzed in peripheral neutrophils of B6 and BTBR mice subjected to methylmercury chloride treatment during their juvenile periods. Our data show that there was a marked increase in the expression of p-NF-kB, iNOS, and nitrotyrosine in the peripheral neutrophils of methylmercury chloride-treated BTBR mice, compared to vehicle BTBR mice treated as shown by an increased percentage of p-NF-kB+, iNOS+, and nitrotyrosine+Gr-1 cells (Figure 4A–C). In addition, methylmercury chloride-treated B6 mice also had a minor increase in NF-kB and oxidative stress markers (iNOS and Nitrotyrosine) than vehicle-treated B6 mice; however, it was not significant (Figure 4A–C). These findings together conclude that the methylmercury chloride treatment during the juvenile period raises the oxidative potential in the peripheral neutrophils of adult BTBR mice.

### 3.4. Effect of Methylmercury Chloride during Juvenile Stage on Nrf2-Mediated Signaling in the Cortex of Adult BTBR and B6 Mice

**Figure 5:**
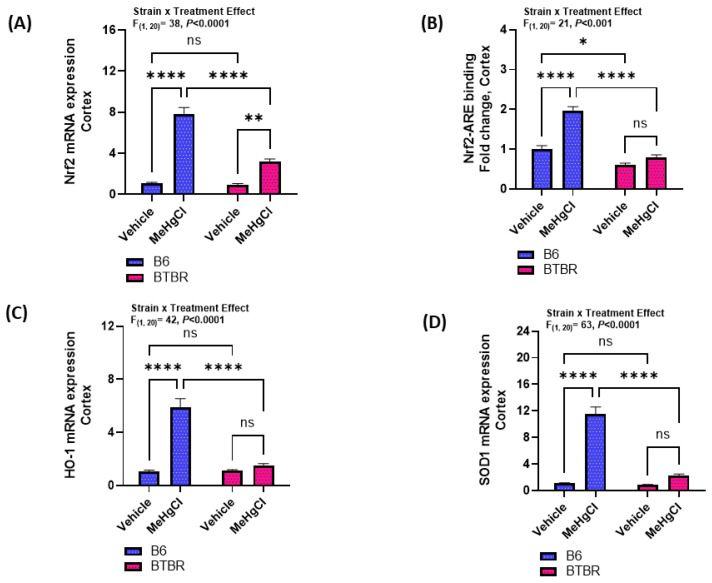
Effect of methylmercury chloride on Nrf2-mediated signaling in the cortex of B6 and BTBR mice. (**A**) Nrf2 mRNA expression, (**B**) Nrf2-ARE binding activity, (**C**) HO-1 mRNA expression, and (**D**) SOD-1 mRNA expression. Values are shown as mean ± SEM; n = 6/group. * *p* < 0.05; ** *p* < 0.01; **** *p* < 0.0001; ns = not significant.

Next, we sought to see how methylmercury chloride affected the Nrf2 signaling and antioxidant enzymes in the cortex of B6 and BTBR mice. Our findings reveal that the Nrf2 mRNA expression increased in both strains after exposure to methylmercury chloride (Figure 5A). However, only adult B6 mice had an increased Nrf2-ARE binding activity in the cortex after exposure to methylmercury chloride during the juvenile stage (Figure 5B). Besides Nrf2-ARE binding activity, a similar increased pattern was observed in the mRNA expressions of HO-1 and SOD-1 in the cortex of B6 and BTBR mice (Figure 5C,D). HO-1 and SOD-1 mRNA expression in the cortex of adult B6 mice had a significant increase after exposure to methylmercury chloride during the juvenile period, whereas there was no effect on these enzymes in BTBR mice (Figure 5C,D). Altogether, these results depict that juvenile exposure to methylmercury chloride treatment fails to augment Nrf2-mediated antioxidant protection in adult BTBR mice.

### 3.5. Effect of Methylmercury Chloride during Juvenile Stage on NF-kB Mediated Signaling in the Cortex of Adult BTBR and B6 Mice

**Figure 6:**
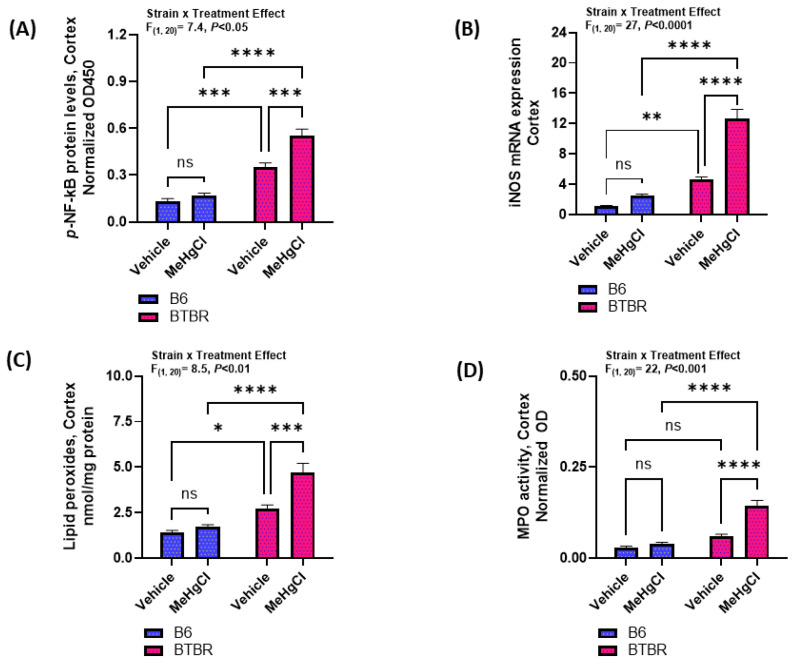
Effect of methylmercury chloride on NFkB-mediated signaling in the cortex of B6 and BTBR mice. (**A**) *p*-NFkB protein levels, (**B**) iNOS mRNA expression, (**C**) Lipid peroxides levels, and (**D**) MPO activity. Values are shown as mean ± SEM; n = 6/group. * *p* < 0.05; ** *p* < 0.01; *** *p* < 0.001; **** *p* < 0.0001; ns = not significant.

The effect of methylmercury chloride on NF-kB levels and oxidative stress markers such as iNOS, lipid peroxidation, and MPO in the cortex of B6 and BTBR mice was also investigated. Our results showed that the cortex of methylmercury chloride-treated BTBR mice had increased levels of p-NF-kB as compared to that of vehicle-treated BTBR mice; however, there was no significant difference in the p-NF-kB levels of the cortex of the vehicle-treated and methylmercury chloride-treated B6 strain (Figure 6A). In addition, markers of oxidative stress, i.e., iNOS and lipid peroxidation in the cortex of adult BTBR mice had a significant increase after exposure to methylmercury chloride during the juvenile period, whereas there was no significant increase in these parameters in adult B6 mice (Figure 6B,C). Further, a marker of neutrophilic inflammation, i.e., MPO, was found to be elevated in the cortex of methylmercury chloride-treated BTBR mice than vehicle-treated BTBR mice; however, there was no significant difference in the cortical MPO levels of vehicle-treated and methylmercury chloride-treated B6 mice (Figure 6D). Overall, these findings reveal that a methylmercury chloride treatment during the juvenile period causes an exacerbation of the autism-like behavior in adult BTBR mice probably due to an increase in oxidative inflammation and failure to upregulate the antioxidant protection.

## 4. Discussion

ASD is characterized by repetitive behaviors and restricted interests, along with barriers to social communication [8]. The pathophysiology of ASD has been linked to dysfunction in several immune cells including neutrophils, microglia, dendritic cells, and T-lymphocytes. Abnormal neuron development, elevated oxidative stress, and the release of pro-inflammatory cytokines can cause neuroinflammation and damage the brain tissue in individuals with autism spectrum disorder [26,27,28,29]. Since the condition is a neurological problem, it is thought that genetic or environmental factors may play a vital role in affecting brain functioning [22,30]. For instance, a study showed that environmental factors account for a 55% probability of having autism spectrum disorder, whereas genetic factors account for only 37% [31]. Over the years, several studies have been conducted that have shown that environmental factors cause alterations in the neuronal function and antioxidant defense system that leads to the development of this neurological disorder [32,33,34,35]. For instance, a recent study shed light on the mechanism of environmental pollutants such as Di-(2-ethylhexyl) phthalate (DEHP) in the aggravation of autism-like behavior in BTBR mice [19]. Therefore, in this study, we aspired to study the effect of another environmental toxicant, i.e., methylmercury chloride, on autism-like behavior in BTBR and B6 mice. The results of our study highlighted that the exposure to methylmercury chloride during the juvenile period displayed exaggerated autism-like behavior in adult BTBR mice.

The activation of peripheral immune cells in various psychiatric disorders including ASD has been extensively studied [7,10,11]. It has been found that peripheral immune cells liberate several inflammation-causing mediators at the blood–brain barrier which impair neural activity via modulating the cellular response in oligodendrocytes, microglia, and astrocytes [25,33,36]. As these inflammatory mediators are considered to augment impairment in neuronal and neuroglial functioning, they may have a crucial role in the pathogenesis of neurodevelopmental impairment linked with ASD [32,36,37]. Previous studies conducted on ASD have shown that peripheral inflammatory mediators, i.e., oxidants and cytokines, mediate the activation of transcription factors (NFkB/Nrf2) and protein kinases in the peripheral immune cells of BTBR mice [33,34,38]. The current study showed that exposure to methylmercury chloride during the juvenile period enhanced the expression of NF-kB/iNOS in adult BTBR mice without substantially balancing the antioxidant response in the cortex and peripheral neutrophils, which led to the aggravation of autism-like symptoms of the BTBR mice. A recent study has also shown that the prenatal exposure of B6 mice to methylmercury at low doses caused autism-like symptoms such as a lack of interaction, repetitive interest, and behavioral variations which was probably due to premature neuronal differentiation [13].

Free radicals are single electronic species that are known to cause oxidative damage to macromolecules such as DNA, proteins, and carbohydrates [28,39,40]. It has been found that during pathological conditions, the level of oxidative stress increases which further aggravates the disease by causing harmful consequences, one of which is the reduction in activity of antioxidant enzymes in the body [41,42]. Furthermore, free radicals and other reactive oxygen species are known to directly damage lipids when present in high concentrations [29] The endoplasmic reticulum, plasma membrane, peroxisomes, and mitochondria are the main sites where endogenous reactive oxygen species are produced [28,43]. Several processes contribute to this process, including enzymatic reactions and/or auto-oxidation of various substances [43]. Oxidative lipid breakdown known as lipid peroxidation generally takes place when free radicals seize electrons from the cell membrane. On the other hand, the oxidation of proteins can lead to the dysfunction of enzymes/receptors [29,42,43]. Hydroxyl radicals, peroxynitrite, superoxide, and hydroperoxyl are the most abundant reactive oxygen species that can have significant effects on lipids and proteins. Several oxidation products, such as lipid hydroperoxides, nitrotyrosine, and malondialdehyde, are generated during protein/lipid oxidation [28,43]. Our study showed increased lipid/protein oxidative products in the neutrophils and cortex of adult BTBR mice which were exposed to a methylmercury treatment during the juvenile period.

Over the past years, the association between oxidative stress and ASD has been intensively studied. For instance, BTBR mice and ASD subjects show upregulation of NF-kB, iNOS, NADPH oxidase, lipid peroxides, and nitrotyrosine which may exacerbate the autism-like symptoms by increasing oxidative damage in the periphery and brain [18,19,27,44,45,46,47,48,49]. Past studies showed that BTBR mice and individuals with ASD had increased lipid peroxidation and other markers of oxidative stress such as nitrotyrosine as compared to normal controls. These oxidative events may exacerbate the autism-like symptoms in mice and human ASD subjects by increasing the oxidative damage in the periphery and brain [43,44,45]. A recent study showed that supplementation of a ketogenic diet alleviated oxidative stress by reducing the level of lipid peroxidation in the brain tissue of BTBR mice which was associated with improvements of autism-like behavior [38]. Other studies also showed that a reduction in peripheral and CNS oxidative stress resulted in the improvement of autism-like symptoms in BTBR mice, whereas an increase in CNS/peripheral oxidative stress caused the worsening of autism-like symptoms in BTBR mice [26,27]. Furthermore, another study showed that the gestational exposure of pregnant BTBR mice to the pesticide chlorpyrifos led to an increase in brain oxidative stress in BTBR pups [46]. Therefore, an increase in oxidative parameters after juvenile exposure to methylmercury may be responsible for the worsening of autism-like symptoms in adult BTBR mice in our study. Our results are consistent with previously published reports showing an association between the upregulation of oxidative stress and ASD [21,27,40,45,46,47,48,49].

The Nrf2 signaling pathway mediates the activation of various antioxidant enzymes, including HO-1 and SOD through the transcriptional regulation of antioxidant response elements in the DNA [50]. Several studies have revealed that a deficiency of Nrf2 in mice significantly enhanced the risk of developing toxicity and oxidative stress-related diseases [50,51]. It is well known that antioxidant defense mechanisms are activated with an increase in oxidative stress, but our results are not consistent with this as adult BTBR mice exposed to methylmercury chloride during the juvenile stage had oxidative stress parameters without the upregulation of Nrf2 signaling as reflected by a lack of HO-1 and SOD-1 upregulation in the periphery and CNS. However, adult B6 mice exposed to methylmercury chloride during the juvenile period showed an upregulation of the Nrf2 signaling pathway and HO-1/SOD-1 antioxidant enzymes which could be responsible for the lack of development of autism-like behavior in these mice. A lack of antioxidant upregulation due to dysregulation in Nrf2 signaling in ASD subjects and BTBR mice has been earlier reported [18,19,27,52]. Considered together, our results confirm that a lack of Nrf2 signaling in adult BTBR mice after exposure to methylmercury during the juvenile stage might be responsible for the worsening of autism-like behavior. Several Nrf2 activators such as resveratrol, sulforaphane, curcumin, and naringenin showed improvements in autistic behavior both in human ASD subjects and mice models of autism which suggests that Nrf2 signaling plays a key role in the amplification of the antioxidant defenses required to protect against oxidant-stress induced by various toxicants/pollutants [27,53,54,55,56].

Our study showed decreased Nrf2-ARE binding in the vehicle-treated BTBR mice as compared to vehicle-treated B6 mice, whereas Nrf2 mRNA levels were not significantly different between the two groups. Nrf2 mRNA levels increased in both groups after methylmercury exposure, which did not translate into increased Nrf2-ARE binding in the BTBR group. It is well known that the translation of an mRNA transcript into a protein might be affected by several factors, e.g., inflammation, toxins, and drugs [57,58]. Therefore, it is feasible that Nrf2 mRNA transcripts were modulated differentially by inflammatory conditions present in the brain of BTBR mice, thereby leading to reduced Nrf2 protein expression and its translocation to the nucleus as reflected by the strong interaction between the strain and treatment (assessed by a two-way ANOVA) in Nrf2-related biochemical parameters.

## 5. Conclusions

In summary, this study provides substantial evidence that the juvenile exposure to methylmercury chloride causes an enhancement of autism-like symptoms in adult BTBR mice possibly due to a paucity in antioxidant defenses and concomitant oxidative damage in the periphery and CNS. Further, it suggests that there is an interaction between genetic background and toxicant exposure during the juvenile stage which may manifest in adulthood. A treatment approach that enhances peripheral and neuronal antioxidants may be a good candidate for preventing environmental toxicant-induced autism-like behaviors and reducing the global morbidity associated with ASD.

## Data Availability

The authors confirm that all data underlying the findings are fully available without restriction. All relevant data are within the paper.
